# Dexmedetomidine hydrochloride inhibits hepatocyte apoptosis and inflammation by activating the lncRNA TUG1/miR-194/SIRT1 signaling pathway

**DOI:** 10.1186/s12950-021-00287-3

**Published:** 2021-05-26

**Authors:** Xiao-Xia Gu, Xiao-Xia Xu, Hui-Hua Liao, Ruo-Na Wu, Wei-Ming Huang, Li-Xia Cheng, Yi-Wen Lu, Jian Mo

**Affiliations:** 1 Department of Anesthesiology, Affiliated Hospital of Guangdong Medical University, No.57, South People’s Avenue, Xiashan District, 524001 Zhanjiang, Guangdong Province P.R. China; 2 Operating room, Affiliated Hospital of Guangdong Medical University, 524001 Zhanjiang, Guangdong Province P.R. China; 3 Department of Thyroid and Breast Surgery, Affiliated Hospital of Guangdong Medical University, 524001 Zhanjiang, Guangdong Province P.R. China

**Keywords:** Dexmedetomidine hydrochloride, TUG1, SIRT1, miR-194, liver injury

## Abstract

**Background:**

Liver injury seriously threatens the health of people. Meanwhile, dexmedetomidine hydrochloride (DEX) can protect against liver injury. However, the mechanism by which Dex mediates the progression of liver injury remains unclear. Thus, this study aimed to investigate the function of DEX in oxygen and glucose deprivation (OGD)-treated hepatocytes and its underlying mechanism.

**Methods:**

In order to investigate the function of DEX in liver injury, WRL-68 cells were treated with OGD. Cell viability was measured by MTT assay. Cell apoptosis was detected by flow cytometry. Inflammatory cytokines levels were measured by ELISA assay. The interaction between miR-194 and TUG1 or SIRT1 was detected by dual-luciferase reporter. Gene and protein levels were measured by qPCR or western blotting.

**Results:**

DEX notably reversed OGD-induced inflammation and apoptosis in WRL-68 cell. Meanwhile, the effect of OGD on TUG1, SIRT1 and miR-194 expression in WRL-68 cells was reversed by DEX treatment. However, TUG1 knockdown or miR-194 overexpression reversed the function of DEX in OGD-treated WRL-68 cells. Moreover, TUG1 could promote the expression of SIRT1 by sponging miR-194. Furthermore, knockdown of TUG1 promoted OGD-induced cell growth inhibition and inflammatory responses, while miR-194 inhibitor or SIRT1 overexpression partially reversed this phenomenon.

**Conclusions:**

DEX could suppress OGD-induced hepatocyte apoptosis and inflammation by mediation of TUG1/miR-194/SIRT1 axis. Therefore, this study might provide a scientific basis for the application of DEX on liver injury treatment.

## Background

Liver injury is a common and serious injury in abdominal trauma. Its incidence ranks second only to spleen rupture, and there are many complications of liver injury [1, 2]. In addition, liver injury usually results from the excessive consumption of alcohol, infections, and xenobiotics, et al. [3]. In addition, it usually leads to liver failure and even death. Nowadays, surgery is the main treatment for liver injury, while the mortality of liver failure has been maintained at a high level [4]. Therefore, it is of great significance to explore the therapeutic targets against liver injury.

Dexmedetomidine hydrochloride (DEX) is a new type of highly selective α-adrenergic receptor agonist that has been widely used in the clinic. It was reported that DEX could inhibit endotoxemia-induced inflammatory response and protect against the organ injury [5]. In addition, DEX is known to alleviate the local and systemic inflammation response by mediation of IL-6 and TNF-α [6]. Furthermore, it had been previously found that DEX could alleviate the severity of hepatic injury [7]. However, the mechanism by which DEX modulates the progression of liver injury remains unclear.

Long non-coding RNAs (lncRNAs) are a class of non-coding RNAs which are over 200 nucleotides in length [8]. A previous study reports that some lncRNAs can play key roles in a variety of diseases, including liver injury [9–11]. In addition, lncRNA taurine up-regulated gene 1 (TUG1) was originally distributed in taurine-treated mouse retinal cells, and it was highly conserved in mammals [12]. A research indicated that TUG1 could regulate the tumor cell growth [13], and TUG1 could attenuate the inflammation during the progression of liver injury [14]. However, the molecular mechanism by which TUG1 attenuates the liver injury is unclear.

MicroRNAs (miRNAs) are a group of small noncoding transcripts which can regulate cell growth and inflammatory responses [15, 16]. Previous studies have found that miRNAs are involved in liver functions. For example, miR-194 could inhibit hepatic stellate cell activation and extracellular matrix production which were associated with liver fibrosis [17]. miR-194 was found to be up-regulated in liver injury and it could inhibit the activity and fibrosis in hepatic stellate cells [18]. Nevertheless, the function of miR-194 in liver injury is largely unknown.

Sirtuin 1 (SIRT1) is a nicotinamide adenine dinucleotide dependent histone deacetylase which plays a crucial role in gene transcription, oxidative stress and inflammation [19]. SIRT1 was down-regulated in doxorubicin-induced liver injury [20]. Meanwhile, upregulation of SIRT1 could protect against the liver injury [21]. In this study, to the data of bioinformatics indicated TUG1 was bound to miR-194 and SIRT1 was a target gene of miR-194. Consequently, it could be hypothesized that DEX protected the hepatocyte injury though regulating TUG1/miR-194/SIRT1 axis in OGD-induced hepatocytes.

In the present study, the function of DEX in hepatocyte apoptosis and inflammation was investigated. Our findings revealed that DEX could attenuate the liver injury by mediation of TUG1/miR-194/SIRT1 signaling pathway. Our study may be provided a theoretical basis for the treatment of liver injury.

## Methods

### Cell culture and drug treatment

The human hepatocytes (WRL-68) were obtained from American Type Culture Collection (ATCC, Manassas, VA, USA). Cells were cultured in RPMI-1640 with 10 % fetal bovine serum (FBS, Invitrogen, Carlsbad, CA, USA) in a condition of 5 % CO_2_ and 95 % humidified air at 37 °C. DEX was obtained from Sigma-Aldrich (catalog number: 1,179,333, St Louis, MO, USA) and diluted in 0.1 % dimethyl sulfoxide (DMSO) (Sigma-Aldrich, St. Louis, MO, USA).

### Oxygen and glucose deprivation (OGD)-induced liver injury

WRL-68 cells were pre-treated with different concentrations of DEX (0, 0.01, 0.1, 1 or 5 nM DEX) for 1 h. Subsequently, cells were cultured in OGD medium (glucose free and serum-free RPMI-1640) under 37 ℃ hypoxia (5 % CO_2_, 1 % O_2_, 94 % N_2_) for 4 h after washed with PBS. Then, cells were reoxygenated (5 % CO_2_, 95 % O_2_) and replaced by conventional medium for 8 h. Meanwhile, cells in control group were cultured in an incubator with 5 % CO_2_ at 37 °C.

### MTT Assay

The cells were seeded in 96-well plates at a density of 2000 cells per well. After 24 h of incubation, the culture medium (200 µL/well) was replaced with FBS-free medium (200 µL/well) containing various concentrations of DEX (0.01, 0.1, 1 and 5 nM). Subsequently, the culture medium was discarded, and 5 mg/mL 3-(4,5-dimethyl thiazol-2-yl)-5-diphenyltetrazolium bromide (MTT) solutions (Sigma, MO, USA) were added in each well for 3 h at 37 °C. Then, the cell supernatants were removed and DMSO (200 µL) was added. The absorbance of each well was measured at 490 nm using a microplate reader (Tecan, Mannedorf, Switzerland).

### Flow cytometry

Apoptotic cells were determined using flow cytometry. Cells were plated in 6-well plates at a density of 2 × 10^5^ cells. After incubation of 48 h, cells were collected with trypsin, washed twice with PBS and centrifuged at 1,500 rpm for 5 min. Subsequently, cells were subjected to apoptosis assay with Annexin V-FITC/PI Apoptosis Detection Kit (BD Pharmingen, Franklin Lake, NJ, USA) according to the instructions of manufacturer. Then, cells were analyzed using a BD Biosciences Fluorescence activated Cell Sorting (FACS, BD Pharmingen, Franklin Lake, NJ, USA) Calibur system with CellQuest Pro software [22].

### Cell transfection

The miR-194 inhibitor, miR-194 mimics, negative control (inhibitor/mimics NC), short hairpin-TUG1 (sh-TUG1) and negative control (sh-NC) were synthetized by Genepharma Inc. (Shanghai, China). For SIRT1 overexpression (pcDNA3.1-SIRT1), the amplified fragment of SIRT1 was inserted into pcDNA3.1 vector (Genepharma Inc., Shanghai, China), and pcDNA3.1 vector was negative control. These plasmids were transfected into cells by using Lipofectamine 2000 (Invitrogen, Carlsbad, CA, USA). Then, cells were cultured with or without DEX for 48 h.

### RNA extraction and RT-qPCR analysis

Total RNA was isolated from cells by using RNA extraction kit (Invitrogen, Carlsbad, CA, USA) according to the manufacturer’s protocol. 2 µg RNA was synthesized into cDNA using SuperScriptTM IV First-Strand Synthesis System (Invitrogen, USA). Real-time qPCR of the reverse transcription products of TUG1, miR-194 and SIRT1 expression was determined using Permix Ex Taq (Takara, Tokyo, Japan), analyzed through the 7500 Real-time PCR System (Applied Biosystems, USA). The data were quantified by normalizing to GAPDH or U6. Relative expression level of genes was quantified using 2^−△△Ct^ method. The primer sequences for qPCR were as follows:

miR-194 F: 5’-TGTAACAGCAACTCCATGTG-3’,

miR-194 R: 5’-GTCGTATCGAGAGCAGGGTCCGAGGTATTCGCACTCGATAC

GACTCCACAT-3’,

SIRT1 F: 5’-CAAACTTTGCTGTAACCCTGT-3’,

SIRT1 R: 5’-CAGCCACTGAAGTTCTTTCAT-3’,

TUG1 F: 5’-TAGCAGTTCCCCAATCCTTG-3’,

TUG1 R: 5’-CACAAATTCCCATCATTCCC-3’,

GAPDH F: 5’-AGGTCGGTGTGAACGGATTTG-3’,

GAPDH R: 5’- GGGGTCGTTGATGGCAACA-3’,

U6 F: 5’-CTCGCTTCGGCAGCACAT-3’,

U6 R: 5’-AACGCTTCACGAATTTGCGT-3’.

### Total protein extraction and Western blot analysis

Total protein was isolated from cell lysates by using RIPA buffer. The concentration of protein was detected with a BCA protein kit (Thermo Fisher Scientific, Waltham, MA, USA). Then, proteins (40 µg per lane) were separated with 10 % sodium dodecyl sulfate-polyacrylamide gel electrophoresis (SDS-PAGE) gel and then transferred into polyvinylidene fluoride (PVDF, Thermo Fisher Scientific) membranes. The membrane was then blocked with 5 % nonfat milk dissolved in TBS buffer for 1 h and incubated with primary antibodies overnight at 4 °C. Subsequently, membranes were incubated with secondary anti-rabbit antibody (1:5000) at room temperature for 1 h. Signals were developed by incubating the membrane with enhanced chemiluminescence and western blot detection reagents, followed by exposure to X-Omat Blue XB-1 film (Kodak, Rochester, NY) for autoradiography. The primary antibodies were as follows: anti-Bax (1:1000), anti-Bcl-2 (1:1000), anti-SIRT1 (1:1000) and anti-GAPDH (1:1000). All the antibodies were obtained from Abcam (Cambridge, MA, USA). The data was analyzed by using ImageJ software.

### Bioinformatics analysis and dual-luciferase reporter gene assay

Starbase (http://starbase.sysu.edu.cn) was used to predict the binding sites between miR-194 and TUG1 or SIRT1. The partial sequences of TUG1 and 3’-UTR of SIRT1 containing the putative binding sites of miR-194 were synthetized and obtained from Sangon Biotech (Shanghai, China), and then cloned into the pmirGLO Dual-Luciferase miRNA Target Expression Vectors (Promega, Madison, WI, USA) for constructing the TUG1 reporter vectors (TUG1-wt/mut) and SIRT1 reporter vectors (SIRT1-wt/mut), respectively. The TUG1-wt/mut vectors or SIRT1-wt/mut vectors were transfected into WRL-68 cells together with mimics NC or miR-194 mimics using Lipofectamine 2000 (Thermo Fisher Scientific). The relative luciferase activity was analyzed by the Dual-Glo Luciferase Assay System (Promega). Renilla luciferase was used as internal control.

### ELISA assay

The levels of IL-1β, IL-6 and TNF-α in the supernatants of WRL-68 cells was determined by ELISA kit (Nanjing Jiancheng Bioengineering Institute, Nanjing, China) according to the instructions of manufacturer. In brief, cells were coated with the primary antibodies overnight, and then blocked with 10 % FBS for 1 h. Subsequently, cells were incubated with the secondary anti-rabbit antibody at room temperature for 1 h. After that, cells were treated with 1 M hydrochloric acid (Beyotime, Shanghai, China). Finally, the OD value was measured by a microreader (450 nm; Thermo Fisher Scientific, Waltham, MA, USA).

### Statistical analysis

All experiments were conducted in triplicate. Data were expressed as the means ± standard deviations (SD) and analyzed using student’s t-test or one-way ANOVA, followed by the Least Significant Difference (LSD) post-hoc test. A *p*-value < 0.05 was considered statistically significant.

## Results

### DEX significantly reversed OGD-induced WRL-68 cell apoptosis and inflammation

**Fig. 1:**
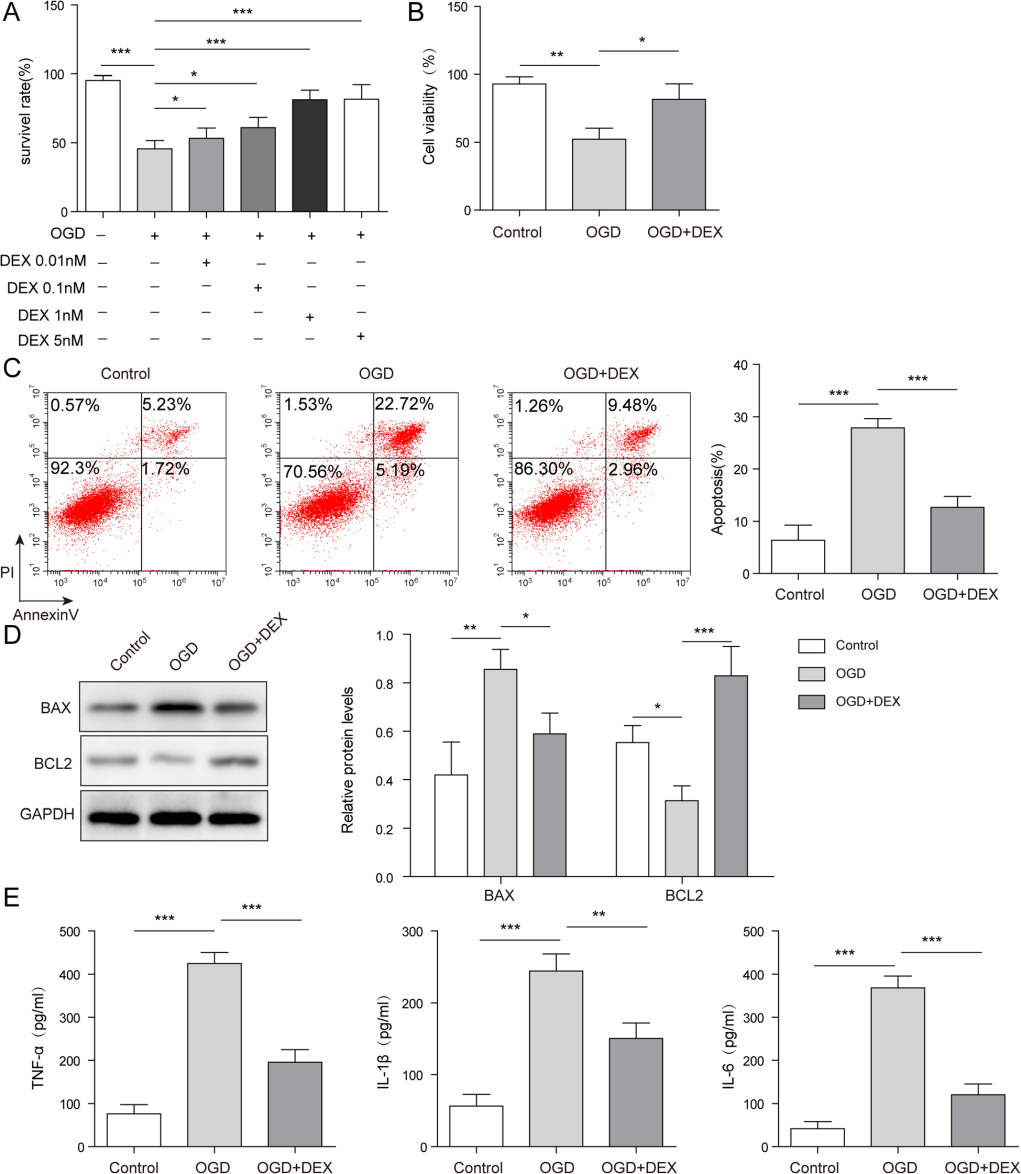
DEX significantly inhibited OGD-induced hepatocyte apoptosis and inflammation. WRL-68 cells were pre-treated with DEX at the concentration of 0, 0.01, 0.1, 1 or 5 nM DEX for 1 h. Then, cells were cultured in OGD medium. **a** The survival rate of WRL-68 cells was tested by MTT assay. **b** Cell viability was tested by MTT assay. **c** The apoptosis of WRL-68 cells was assessed by flow cytometry. **d** The protein expressions of Bcl-2 and Bax in WRL-68 cells were tested by western blotting. The relative protein expressions were quantified via normalizing to GAPDH. **e **The levels of inflammatory cytokines (IL-6, IL-1β and TNF-α) in supernatants of WRL-68 cells were assessed by ELISA. *p < 0.05, ** *p* < 0.01 and *** *p* < 0.001

To test the cell viability, MTT assay was performed. As indicated in Fig. 1 a, the survival rate of WRL-68 cells was significantly decreased by OGD treatment, while DEX reversed this phenomenon in a dose-dependent manner. Moreover, DEX reversed OGD-induced inhibition of cell viability (Fig. 1b). Meanwhile, OGD notably induced the apoptosis of WRL-68 cells, while the apoptotic effect of OGD was partially rescued in the presence of DEX (Fig. 1 c). Consistently, OGD treatment significantly inhibited the expression of Bcl-2 but increased the level of Bax in WRL-68 cells, while the effect of OGD on these two proteins was partially reversed by using DEX (Fig. 1d). Furthermore, the levels of IL-1β, IL-6, TNF-α in supernatants of WRL-68 cells were increased by OGD, which was significantly rescued by DEX (Fig. 1e). Taken together, DEX inhibited the OGD-induced cell growth inhibition and inflammatory responses.

### DEX reversed the expressions of TUG1, miR-194 and SIRT1 in WRL-68 by OGD-induced

**Fig. 2:**
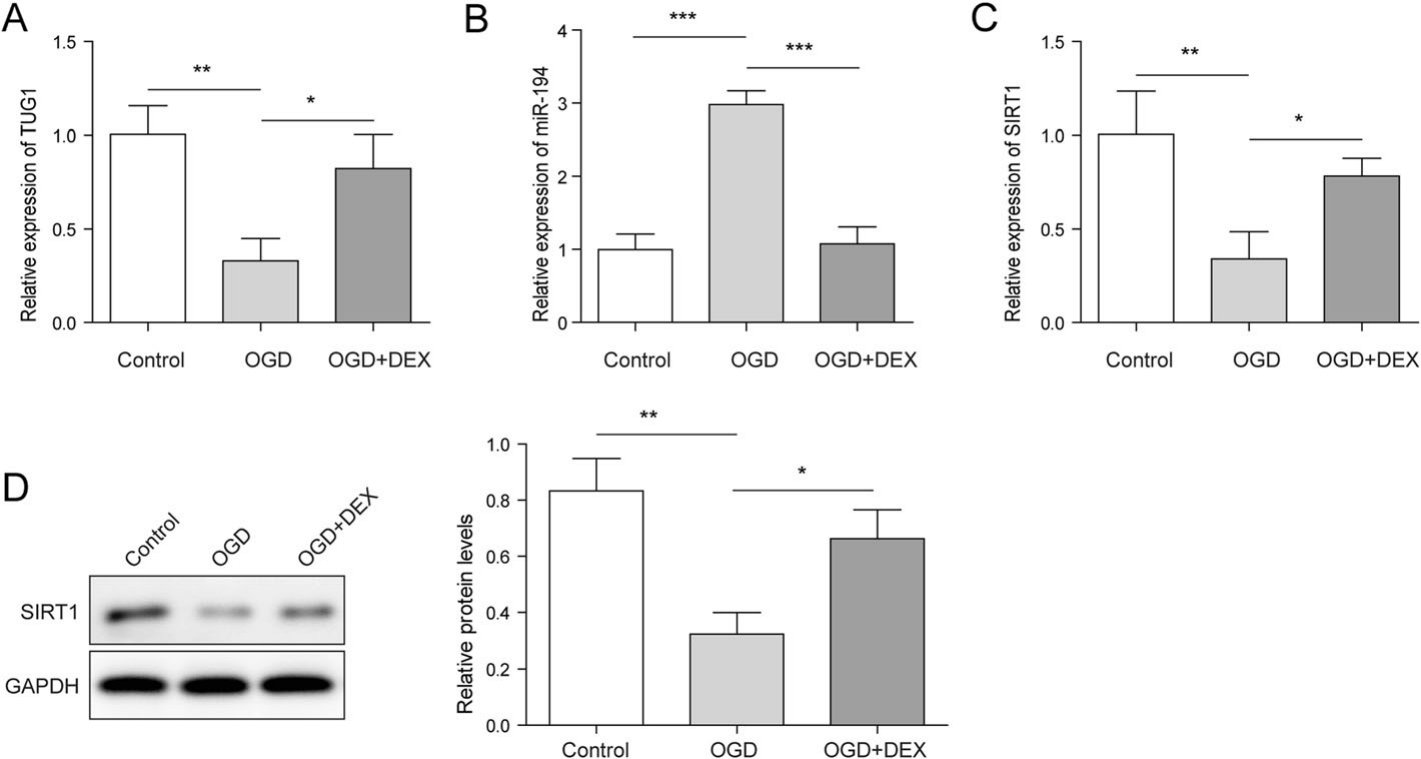
DEX reversed the expressions of TUG1, miR-194 and SIRT1 in WRL-68 by OGD-induced. WRL-68 cells were pre-treated with 1 nm DEX for 1 h and then cultured in OGD medium. **a** The expression of TUG1 in WRL-68 cells was detected by qPCR assay. **b** The level of miR-194 in WRL-68 cells was detected by qPCR assay. **c** The level of SIRT1 in WRL-68 cells was detected by qPCR assay. **d** The protein expression of SIRT1 in WRL-68 cells was detected by western blotting. The relative protein expressions were quantified via normalizing to GAPDH. *p < 0.05, ** *p* < 0.01 and *** *p* < 0.001

As shown in Fig. 2 a and Fig. 2 c, the expressions of TUG1 and SIRT1 in WRL-68 cells were down-regulated by OGD treatment, while this phenomenon was reversed by DEX. In contrast, OGD-induced upregulation of miR-194 was significantly rescued in the presence of DEX (Fig. 2b). Consistently, DEX significantly reversed the effect of OGD on SIRT1 expression (Fig. 2d). To sum up, DEX might inhibit OGD-induced hepatocyte injury via mediation of TUG1, SIRT1 and miR-194.

### The protective effect of DEX against hepatocyte injury was reversed by TUG1 knockdown

**Fig. 3:**
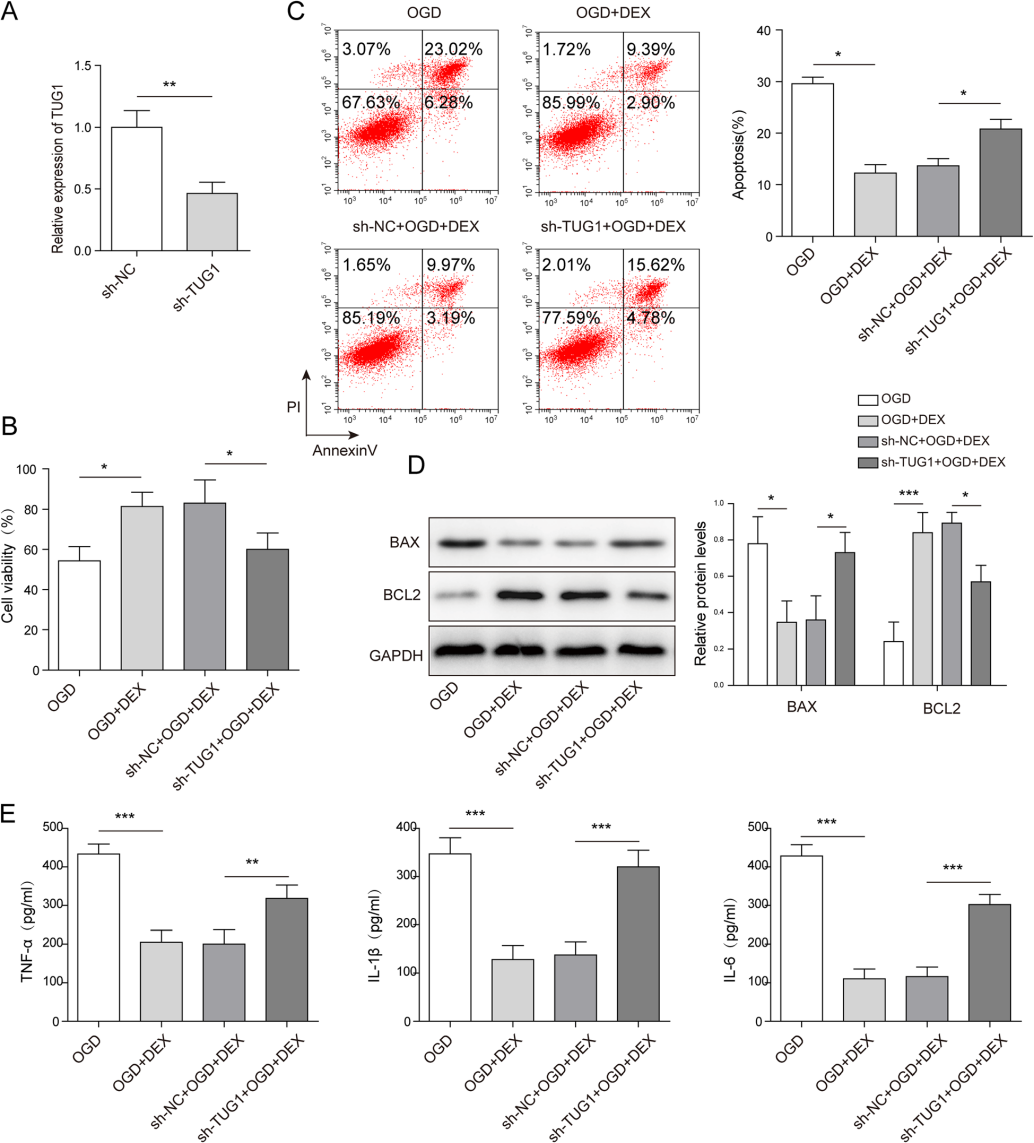
TUG1 knockdown reversed DEX-induced inhibition of inflammatory responses and apoptosis in OGD-treated hepatocytes. WRL-68 cells were pre-treated with 1 nm DEX for 1 h and then cultured in OGD medium. **a** The expression of TUG1 was detected by qPCR. **b** Cell viability was tested by MTT assay. **c** The apoptosis of WRL-68 cells was assessed by flow cytometry. **d** The protein expressions of Bcl-2 and Bax in WRL-68 cells were tested by western blotting. The relative protein expressions were quantified via normalizing to GAPDH. **e** The levels of inflammatory cytokines (IL-6, IL-1β and TNF-α) in supernatants of WRL-68 cells were assessed by ELISA. *p < 0.05, ** *p* < 0.01 and *** *p* < 0.001

To investigate the correlation between DEX and TUG1 in OGD-induced hepatocyte injury, the qPCR and MTT assay were performed. As indicated in Fig. 3 a, the expression of TUG1 in WRL-68 cells was obviously inhibited by TUG1 shRNA. This data suggested that TUG1 shRNA was successfully transfected into WRL-68 cells. In addition, the viability of OGD-treated WRL-68 was increased by DEX, while TUG1 knockdown notably reversed this phenomenon (Fig. 3b). Moreover, silencing of TUG1 significantly reversed the inhibitory effect of DEX on apoptosis of OGD-treated WRL-68 cells (Fig. 3 c). Furthermore, DEX significantly reversed the effect of OGD on Bax and Bcl-2 expressions, while TUG1 shRNA partially rescued this phenomenon (Fig. 3d). Meanwhile, the levels of IL-1β, IL-6, TNF-α in supernatants of OGD-treated WRL-68 cells were greatly inhibited by DEX, while the anti-inflammatory effect of DEX was partially restored by TUG1 shRNA (Fig. 3e). All these results indicated that the effect of DEX on hepatocyte injury was reversed by TUG1 knockdown.

### TUG1 mediated miR-194/SIRT1 axis in WRL-68 cells

**Fig. 4:**
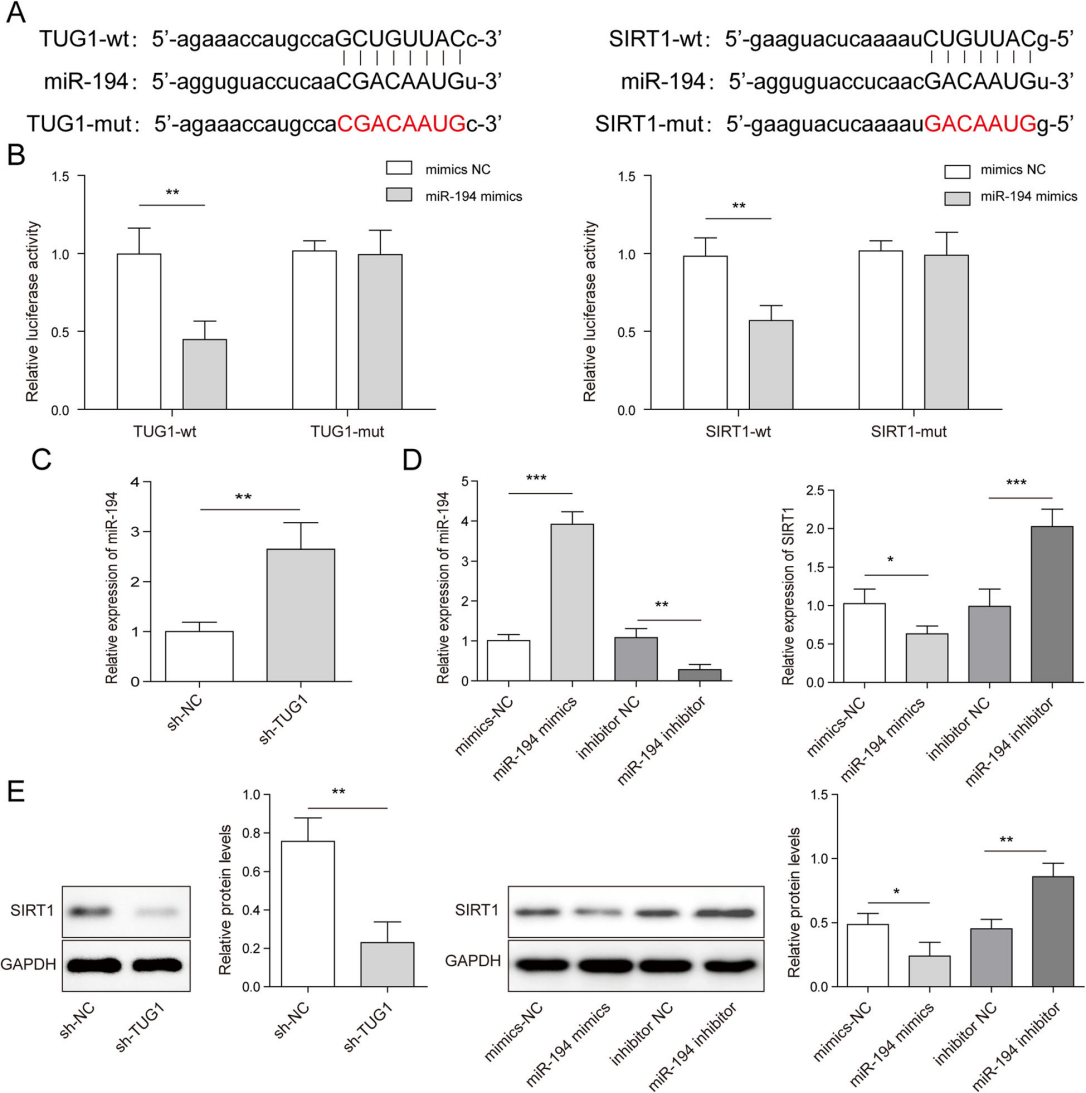
TUG1 mediated miR-194/SIRT1 axis in hepatocytes. **a** Bioinformatics analysis was used to predict the bind sites between miR-194 and TUG1 or SIRT1. **b** The relative luciferase activity was tested by dual-luciferase reporter gene assay. WRL-68 cells were pre-treated with 1 nm DEX for 1 h and then cultured in OGD medium. **c** The expression of miR-194 in WRL-68 cells was assessed by qPCR. **d** The expressions of miR-194 and SIRT1 in WRL-68 cells were assessed by qPCR. **e** The protein expression of SIRT1 in WRL-68 cells was tested by western blotting. The relative protein expressions were quantified via normalizing to GAPDH. *p < 0.05, ** *p* < 0.01 and *** *p* < 0.001

In order to investigate the mechanism by which TUG1 mediates the progression of OGD-treated hepatocyte injury, bioinformatics analysis was performed. The data suggested that TUG1 had putative binding sites with miR-194, and miR-194 might directly target SIRT1 (Fig. 4 a). For further verification, dual-luciferin reporter gene assay was applied. As shown in Fig. 4b, the relative luciferase activity was decreased by miR-194 mimics co-transfected with TUG1-wt or SIRT1-wt, whereas no significant difference was observed with TUG1-mut or SIRT1-mut, suggesting that there were potential binding sites between miR-194 and TUG1, and SIRT1 would be a target gene of miR-194. Furthermore, the qPCR results showed that sh-TUG1 could up-regulate the expression of miR-194 (Fig. 4 c). Then, the expression of miR-194 was increased by miR-194 mimics but reduced in the presence of miR-194 inhibitor, and miR-194 could negatively regulate SIRT1 level (Fig. 4d). Moreover, western blotting results showed that miR-194 mimics and sh-TUG1 could inhibit the expression of SIRT1 (Fig. 4e). Thereby, the data suggested that TUG1 regulated SIRT1 expression via sponging miR-194.

### MiR-194 mimics significantly reversed DEX-induced inhibition of OGD-induced WRL-68 cell injury

**Fig. 5:**
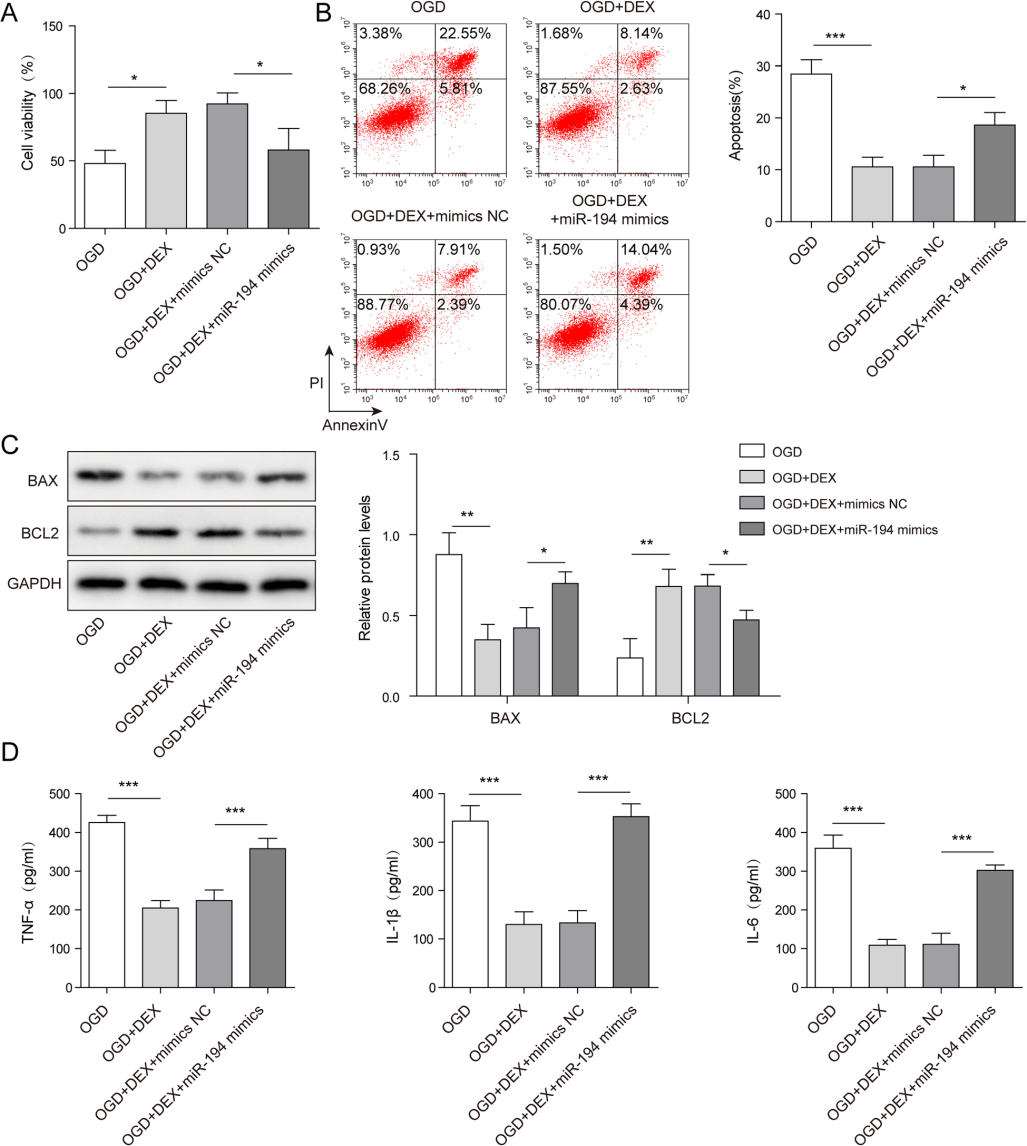
The protective effect of DEX against OGD-induced WRL-68 cell injury was reversed by miR-194 mimics. WRL-68 cells were pre-treated with 1 nm DEX for 1 h and then cultured in OGD medium. **a** Cell viability was assessed by MTT assay. **b** The apoptosis of WRL-68 cells was assessed by flow cytometry. **c** The protein expressions of Bcl-2 and Bax in WRL-68 cells were tested by western blotting. The relative protein expressions were quantified via normalizing to GAPDH. **d** The levels of inflammatory cytokines (IL-6, IL-1β and TNF-α) in supernatants of WRL-68 cells were assessed by ELISA. *p < 0.05, ** *p* < 0.01 and *** *p* < 0.001

In order to detect the function of miR-194 in DEX-mediated hepatocyte injury, MTT was performed. As shown in Fig. 5 a, DEX could increase the viability of OGD-treated WRL-68 cells, while the effect of DEX on cell viability was rescued in the presence of miR-194 mimics. Consistently, miR-194 mimics reversed the anti-apoptotic effect of DEX on OGD-treated hepatocytes (Fig. 5b). In addition, DEX significantly up-regulated the level of Bcl-2 but inhibited the expression of Bax in OGD-treated WRL-68 cells, which was partially reversed by miR-194 mimics (Fig. 5 c). Furthermore, DEX-induced decrease of TNF-α, IL-1β and IL-6 in OGD-treated WRL-68 cells was rescued in the presence of miR-194 mimics (Fig. 5d). In summary, miR-194 mimics reversed the protective effect of DEX against OGD-induced WRL-68 cell injury.

### DEX inhibited OGD-induced hepatocyte injury via mediation of TUG1/miR-194/SIRT1 axis

**Fig. 6:**
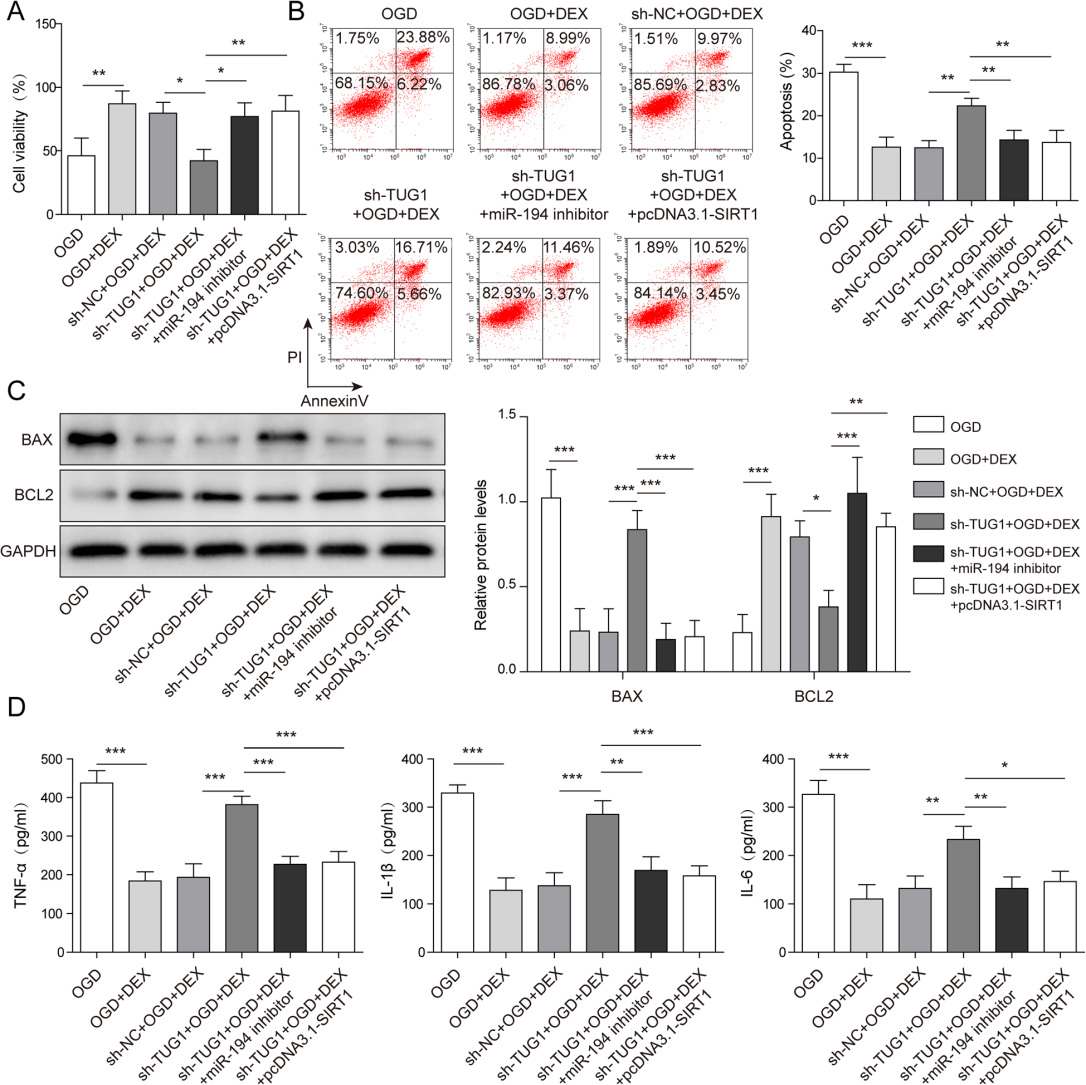
DEX inhibited OGD-induced hepatocyte injury via mediation of TUG1/miR-194/SIRT1 axis. WRL-68 cells were pre-treated with 1 nm DEX for 1 h and then cultured in OGD medium. (A) Cell viability was detected by MTT assay. (B) The apoptosis of WRL-68 cells was assessed by flow cytometry. (C) The protein expressions of Bcl-2 and Bax in WRL-68 cells were tested by western blotting. The relative protein expressions were quantified via normalizing to GAPDH. (D) The levels of inflammatory cytokines (IL-6, IL-1β and TNF-α) in supernatants of WRL-68 cells were assessed by ELISA. *p < 0.05, ** *p* < 0.01 and *** *p* < 0.001

To further confirm the mechanism by which DEX mediates OGD-induced hepatocyte injury, MTT assay was used. As shown in Fig. 6 a, DEX could reverse the effect of OGD on cell viability, while this phenomenon was partially reversed by TUG1 silencing. However, the effect of TUG1 knockdown was reversed by miR-194 inhibition or SIRT1 overexpression **(**Fig. 6 a**)**. In addition, TUG1 shRNA could reverse DEX-induced inhibition of cell apoptosis, which was rescued by miR-194 inhibition or SIRT1 up-regulation (Fig. 6b). Consistently, knocking down TUG1 partially inhibited the effect of DEX on Bax and Bcl-2 expressions in OGD-treated WRL-68 cells, while miR-194 inhibitor or pcDNA3.1-SIRT1 reversed this phenomenon (Fig. 6 c). Finally, silencing of TUG1 up-regulated the levels of TNF-α, IL-1β and IL-6 in DEX and OGD co-treated cell supernatants (Fig. 6d). Nevertheless, the effect of TUG1 shRNA on these inflammatory factors in hepatocytes was reversed when transfected with miR-194 inhibitor or pcDNA3.1-SIRT1 (Fig. 6d). Taken with the above data, DEX inhibited OGD-induced hepatocyte injury by mediation of TUG1/miR-194/SIRT1 axis.

## Discussion

Prolonged portal vein occlusion can lead to ischemic liver injury in liver transplantation. If the blood supply is restored quickly, cells can be protected against injury. However, improper reperfusion impairs the functions of organs, including liver [23–26]. Ischemia has become a serious obstacle to liver transplantation. It can induce oxygen and nutrient deprivation [27]. The present study firstly found that DEX could reverse OGD-induced hepatocyte injury through regulation of TUG1/miR-194/SIRT1 signaling pathway, further supplementing the mechanism by which DEX mediated the protection against of liver injury.

It has been previously reported that DEX can act as an anti-inflammatory agent [28, 29]. For instance, Feng Z et al. found DEX attenuated the inflammatory responses in fatty liver disease [30]. In addition, DEX reduced ventilator-induced inflammation in lung injury via ERK1/2 pathway activation [31]. Meanwhile, it wasreported that DEX could inhibit the liver injury. Tong F et al. showed that DEX alleviated lipopolysaccharide induced acute liver injury in rats by inhibiting caveolin-1 downstream signaling pathway [32]. Zhou H et al. found that DEX could inhibit the liver injury though promoting macrophage M2 activation via PPARγ/STAT3 signaling [33]. Consistently, our data indicated that DEX reversed OGD-induced inflammatory responses and injury in hepatocytes, suggesting that DEX could act as an inhibitory agent in liver injury. On the other hand, DEX ameliorated liver injury *in vivo* through α2A subtype, and the mechanism was due to inhibit TLR4/NF-κB pathway and reduce the level of inflammatory mediators [34]. Consistently, our study found that DEX suppressed the inflammatory responses in OGD-treated hepatocytes through mediation of TUG1/miR-194/SIRT1 axis. Thus, the protective functions of DEX on liver injury could regulate the different mechanisms between *in vitro* and *in vivo* study. Intriguingly, it was reported that 30 µg/kg improved LPS-induced acute liver injury in SD rats [35]. Saleh et al. found that continuous use of 0.8 µg/kg/h DEX during the operation could protect the ischemia-reperfusion injury and enhance the liver function after adult liver transplantation [36]. However, in our study indicated that 1 nm DEX inhibited ODG-induced hepatocytes proliferation and promoted apoptosis, and this concentration also used in many researches [37].

It has been reported that TUG1 plays important roles in cell injury and inflammation [38, 39]. In addition, TUG1 has been found to relieve the liver injury [40]. Our finding was consistent to this previous report, confirming that TUG1 could serve as a mediator in liver injury. Meanwhile, our study found that miR-194/SIRT1 axis could be regulated by TUG1, while Zhang H et al. indicated that TUG1 could inhibit LPS-induced apoptosis and inflammatory response though down-regulation of miR-29b, NF-κB and JAK/STAT pathways [14]. In our study, we found that DEX increased TUG1 expression in OGD-induced WRL-68, however, knockdown of TUG1 reversed the protective effect of DEX in OGD-induced WRL-68 cells, suggesting DEX played a key role in liver injury by regulating the expression of TUG1. Thus, more mechanisms by which TUG1 regulates liver injury need to be explored in the future.

Recently, serum liver-cell-derived microRNAs (HDmiRs) were considered as early, stable, sensitive and specific biomarkers for liver injury [41]. Meanwhile, miR-194 is known to be a crucial mediator in inflammatory responses [42, 43]. Moreover, miR-194 could aggravate the liver injury [44]. Consistently, this research found that miR-194 was up-regulated in OGD-treated hepatocyte injury, and miR-194 overexpression restored the regulatory effect of DEX on apoptosis and inflammation in OGD-induced WRL-68 cells. In addition, miR-194 was overexpressed in patients with liver diseases, and Yangonin inhibited cell aging by regulating miR-194 and alleviated alcohol-induced liver injury [44]. Our research was similar to this study, indicating that miR-194 could be sponged by TUG1 in hepatocyte injury. Taken with the above results, miR-194 could act as an important mediator of liver function.

SIRT1, an NAD^+^ dependent type III histone/protein deacetylase, plays a key role in protecting against cellular stress and in controlling metabolic pathways which are critical to ischemia/hypoxia [45, 46]. In addition, previous studies found SIRT1 inhibited inflammatory responses in heart, liver, brain and kidney injury [47, 48]. Meanwhile, our research indicated that SIRT1 overexpression could reverse the pro-inflammatory effect of sh-TUG1 in hepatocytes. Thus, our data were in line with the function of SIRT1, confirming that TUG1 mediated the inflammatory responses via indirectly targeting SIRT1. On the other hand, NLRP3 is known to be an inflammasome, and it can promote the levels of inflammatory cytokines in organ respiration [49, 50]. It was found that SIRT1 inhibited the activation of NLRP3 and the secretion of IL-1β in cerebral ischemia [51]. In present study, our findings revealed that SIRT1 could act as an inflammatory suppressor in progression of hepatocytes injury. Taken together, DEX could inhibit OGD-induced hepatocyte apoptosis and inflammation via regulating of TUG1/miR-194/SIRT1 axis.

## Conclusions

In the present study, *in vitro* data showed that DEX could protect against OGD-induced hepatocyte injury. In addition, DEX reversed OGD-induced hepatocyte apoptosis and inflammation through regulation of TUG1, miR-194 and SIRT1. The protective effect of DEX on OGD-treated hepatocytes was accomplished through TUG1/miR-194/SIRT1 signaling pathway. These results might provide a scientific basis for the application of DEX on treatment of liver injury. However, more randomized controlled trials were required to verify the effect of DEX in clinic.

## Data Availability

All data generated or analyzed during this study are included in this article. The datasets used and/or analyzed during the current study are available from the corresponding author on reasonable request.

## Abbreviations

DEX: Dexmedetomidine hydrochloride
OGD: Oxygen-glucose deprivation
TUG1: Taurine up-regulated gene 1
LncRNAs: Long non-coding RNAs
SIRT1: Sirtuin 1
SDS-PAGE: Sodium dodecyl sulfate-polyacrylamide gel electrophoresis
PVDF: Polyvinylidene fluoride
DMSO: Dimethyl sulfoxide
FACS: Fluorescence activated Cell Sorting
LSD: Least significant difference
MTT: 3-(4,5-dimethyl thiazol-2-yl)-5-diphenyl tetrazolium bromide
NC: Negative control
SD: Standard deviations
